# Expression levels of circulating miRNAs as biomarkers during multimodal treatment of rectal cancer - TiMiSNAR-mirna: a substudy of the TiMiSNAR Trial (NCT03962088)

**DOI:** 10.1186/s13063-020-04568-9

**Published:** 2020-07-25

**Authors:** Igor Monsellato, Elisabetta Garibaldi, Elisa Cassinotti, Ludovica Baldari, Luigi Boni, Ugo Elmore, Roberto Delpini, Riccardo Rosati, Roberto Perinotti, Filippo Alongi, Elisa Bertocchi, Stefania Gori, Giacomo Ruffo, Graziano Pernazza, Fabio Pulighe, Carlo De Nisco, Emilio Morpurgo, Tania Contardo, Enzo Mammano, Federico Perna, Benedetta Menegatti, Andrea Coratti, Piero Buccianti, Riccardo Balestri, Cristina Ceccarelli, Davide Cavaliere, Leonardo Solaini, Giorgio Ercolani, Elena Traverso, Vittorio Fusco, Valter Torri, Sara Orecchia

**Affiliations:** 1 Department of Surgery, Azienda Ospedaliera SS. Antonio e Biagio e Cesare Arrigo, Via Venezia 16, 15121 Alessandria, Italy; 2 Department of Radiation Oncology, Azienda Ospedaliera SS. Antonio e Biagio e Cesare Arrigo, Alessandria, Italy; 3 Department of Surgery, Fondazione IRCCS Ca’ Granda, Ospedale Maggiore Policlinico, University of Milan, Milan, Italy; 4 Ospedale San raffaele IRCCS, Milan, Italy; 5 Ospedale degli Infermi, Biella, Italy; 6 Ospedale Sacro Cuore Don Calabria, Negrar, Italy; 7 Azienda Ospedaliera San Giovanni Addolorata, Rome, Italy; 8 Ospedale San Francesco, Nuoro, Italy; 9 Ospedale Civile Pietro Cosma, Camposampiero/Ospedale Sant’Antonio, Padova, Italy; 10 Ospedale Civile Pietro Cosma, Camposampiero, Padova, Italy; 11 Azienda Ospedaliero Universitaria Careggi, Florence, Italy; 12 Azienda Ospedaliero Universitaria Pisana, Pisa, Italy; 13 Ospedale “G.B. Morgagni L. Pierantoni”, Forlì, Italy; 14 Department of Oncology, Azienda Ospedaliera SS. Antonio e Biagio e Cesare Arrigo, Alessandria, Italy; 15 Istituto di Ricerche Farmacologiche Mario Negri IRCCS, Milan, Italy; 16 Department of Pathology, Azienda Ospedaliera SS. Antonio e Biagio e Cesare Arrigo, Alessandria, Italy

**Keywords:** Colorectal cancer, miRNA, Neoadjuvant treatment, Biomarkers, Translational research

## Abstract

**Background:**

Neoadjuvant chemoradiotherapy followed by surgery is the mainstay treatment for locally advanced rectal cancer, leading to significant decrease in tumor size (downsizing) and a shift towards earlier disease stage (downstaging). Extensive histopathological work-up of the tumor specimen after surgery including tumor regression grading and lymph node status helped to visualize individual tumor sensitivity to chemoradiotherapy, retrospectively. As the response to neoadjuvant chemoradiotherapy is heterogeneous, however, valid biomarkers are needed to monitor tumor response. A relevant number of studies aimed to identify molecular markers retrieved from tumor tissue while the relevance of blood-based biomarkers is less stringent assessed. MicroRNAs are currently under investigation to serve as blood-based biomarkers. To date, no screening approach to identify relevant miRNAs as biomarkers in blood of patients with rectal cancer was undertaken. The aim of the study is to investigate the role of circulating miRNAs as biomarkers in those patients included in the TiMiSNAR Trial (NCT 03465982). This is a biomolecular substudy of TiMiSNAR Trial (NCT03962088).

**Methods:**

All included patients in the TiMiSNAR Trial are supposed to undergo blood collection at the time of diagnosis, after neoadjuvant treatment, after 1 month from surgery, and after adjuvant chemotherapy whenever indicated.

**Discussion:**

TiMiSNAR-MIRNA will evaluate the association of variation between preneoadjuvant and postneoadjuvant expression levels of miRNA with pathological complete response. Moreover, the study will evaluate the role of liquid biopsies in the monitoring of treatment, correlate changes in expression levels of miRNA following complete surgical resection with disease-free survival, and evaluate the relation between changes in miRNA during surveillance and tumor relapse.

**Trial registration:**

Clinicaltrials.gov NCT03962088. Registered on 23 May 2019.

## Background

Neoadjuvant chemoradiotherapy (nCHT) followed by surgery is the main treatment for locally advanced rectal cancer, leading to significant decrease in tumor size (downsizing) and a shift towards earlier disease stage (primary tumor and lymph nodes involvement—downstaging) [1]. As the response to nCHT is heterogeneous, however, valid biomarkers are needed to monitor tumor response [2–4]. Therefore, it is of high importance to stratify and identify those patients, who can benefit from an individualized targeted therapy. To date, a significant number of studies aims to identify molecular markers retrieved from tumor tissue while the relevance of blood-based biomarkers is less stringent assessed.

Blood samples, i.e., liquid biopsy, indeed, offer several advantages [5, 6]:

1. Taking blood samples is less invasive, less expensive, easy to schedule, and nearly without any severe complications.
2. Blood samples are a source of fresh DNA and RNA, without modifications due to preservatives; especially in the case of rectal cancer, beyond intratumoral heterogeneity, tumor biopsies are in general accompanied by normal, adenomatous, or stromal tissue. This contamination may affect results of molecular analyses
3. Investigating blood from patients can account for molecular heterogeneity and surrogate for tumor burden since tumor-derived fragments or biomarkers are collected from all tumor cells in a patients’ body through circulation.
4. Liquid biopsy may offer both the possibility of dynamic monitoring under treatment and the possibility to assess disease activity even after pathologic complete response (pCR) or after resection of the tumor when no tissue is left for molecular analyses.

Carcinoembryonic antigen (CEA) is, to date, established as a colorectal cancer (CRC)-related tumor marker, but its unsuitability as a screening and prognostic marker has been demonstrated [7]. Circulating tumor DNA (ctDNA) represents, nowadays, the main approach to monitor tumor burden and therapy resistance, to evaluate the presence of residual disease after potentially curative treatment, and to monitor disease recurrence with high sensitivity and specificity [8].

MicroRNAs (miRNAs) are currently under investigation to serve as blood-based biomarkers as a potential alternative to CEA and ctDNA. miRNAs are small, noncoding RNAs that regulate gene expression by post-transcriptional mRNA binding, which promotes the destabilization of target miRNAs. They are highly conserved between species, stable, and easily detectable even in small concentrations and have been widely analyzed in physiological and pathological processes, and their expression is tissue specific [9–11]. miRNA genes often have multiple transcription start sites, and the promoters of intronic miRNAs are sometimes distinct from the promoters of their host genes [11, 12]. miRNA biogenesis process follows two steps: a nucleic and a cytoplasmatic phase. In nucleus, miRNAs are transcribed in primary-microRNAs by RNA polymerase II and this process is controlled by RNA Pol II-associated transcription factors and epigenetic regulators [12, 13]. Further, they are processed by Drosha RNase III endonuclease in shorter stem loops of about 60–70 nucleotides in length, called pre-miRNAs [13]. Pre-miRNAs are then transported from the nucleus to the cytoplasm via Exportin 5 and processed in mature miRNAs by RNase III endonuclease Dicer [14–17]. Further, maturation of miRNAs is carried out by the RISC-loading complex (RNA-induced silencing complex) [18]. miRNAs constitute the largest class of gene regulation and are involved in all developmental processes, including stem cell and germline maintenance, development and differentiation, transcriptional and post-transcriptional gene silencing, and subcellular localization [19, 20]. miRNAs regulate gene expression through the degradation of mRNA transcripts of their target genes and the translation regulation of mRNA transcript without RNA degradation [21].

Expression patterns of miRNA can be developmental stage specific or, in other circumstances, tissue and site specific. The target specificity of miRNAs is largely predetermined by their so-called seed-sequence (containing nucleotides at positions 2–7 of the miRNA).

It is well-known that miRNA is present in blood, but its lability and the presence of ribonuclease in the plasma raised some questions about how miRNA is carried in the blood flow and its detectability [22, 23], suggesting a mechanism of protection against ribonuclease degradation.

One of the protecting mechanisms that have been suggested is that extracellular RNA is bound with DNA [24], but it has been excluded afterwards [25]. Another mechanism of RNA release that has been at first postulated was cell death like apoptosis or mechanical stress in which apoptotic bodies [26] containing miRNAs are released in the blood flow, thus protecting them from Rnase degradation [27]. It is well-known also that most cell types release continuously soluble factors and exfoliate membrane-derived vesicles into the extracellular space [28]. These kind of vesicles are called exosomes and are distinctly different from apoptotic bodies. Exosomes are nanovesicles that are involved in cell-to-cell communication and regulation of different biological processes [29]. In recent years, exosomes have emerged being involved in both physiological processes, such as immune response and neuronal function, and also in the development and progression of disease, such as cancer [30–32].

Exosomes can facilitate intercellular communication through transportation of grown factors and miRNAs and other small molecules, constituting the probable mechanism of miRNA transportation and protection against degradation [33, 34]. RNA has been found, indeed, on cancer cell surface, and it has been also found in vesicles shed in vitro from a human colon adenocarcinoma cell line [35]. Cancer cells have been demonstrated to secrete high quantity of exosomes than normal cells [33, 34], and exosomal miRNAs are supposed to play an important role in cancer cell proliferation, angiogenesis, metastasis, drug resistance, and tumor inhibition [36–41]; some studies have shown the role of exosomal miRNAs in cellular pathways from life to death, from metabolism to communication [35]. Phenotypes of tumors, indeed, have been demonstrated depending not only on cancer cells but also on surrounding tumor microenvironment [42]. Cancer-cell derived exosomes-miRNAs contribute to the recruitment and reprogramming of constituents associated with tumor environment, modifying the extracellular matrix, reprogram functions of immunologically active factor and immune target cells [43]. To date, four mechanisms are known through which miRNAs influence tumor microenvironment: (1) self-modulation through which less aggressive cancer cells receive exosomal miRNAs delivered by more aggressive cancer cells [44, 45]; (2) distant communication with other cells in the tumor microenvironment for preparing a distant site of tumor proliferation (metastasis-inducing mechanism by downregulation of tight junctions and endothelial monolayers destruction) [46, 47]; (3) miRNAs from normal cells that can alter the behavior of tumor cells [48]; and (4) viral infection that stimulates secretion of exosomes with aberrant miRNAs inducing normal cells in a pre-tumoral condition [49].

Based on these findings, miRNA detection in plasma can play a crucial prognostic role from initial to developmental phase of tumorigenesis and tumor progression, with a fascinating possibility for personalized tumor therapy [50, 51].

To date, no screening approach to identify relevant miRNAs as biomarkers in blood of patients with rectal cancer has been undertaken.

## Methods/design

The Timing To Minimally Invasive Surgery After Neoadjuvant Chemoradiotherapy For Rectal Cancer: A Multicenter Randomized Controlled Trial - Biomarkers Substudy is an observational prospectically design study on the evaluation of the circulating miRNA in serum.

**Fig. 1:**
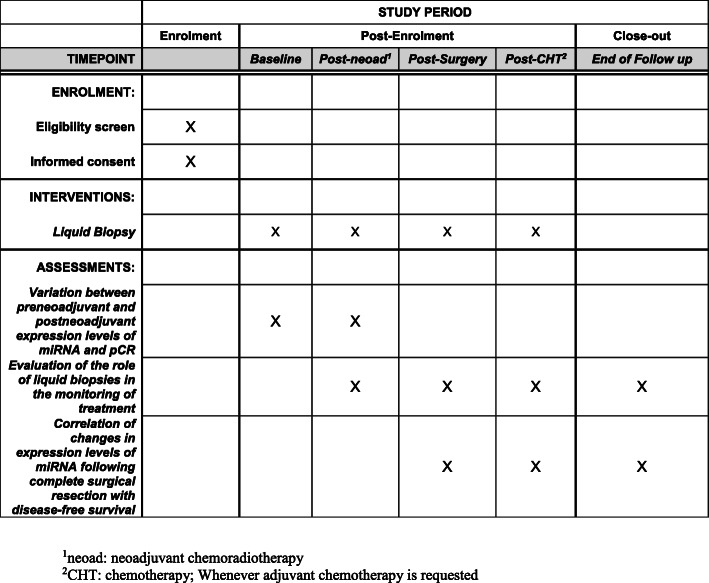
SPIRIT figure

All included patients in the TiMiSNAR Trial (already approved by local Ethical Committees on 8/5/2018) are supposed to undergo blood collection at the time of diagnosis, 1 month after neoadjuvant treatment, 1 month after surgery, and at 1, 3, 6, and 12 months during adjuvant chemotherapy (based on therapy protocol), whenever indicated or at 1, 3, 6 and 12 months during surveillance (Fig. 1).

An informed consent to participate has been prepared and will be obtained by all the participants and collected by the Principal investigator (Dr. Igor Monsellato). Vacutainer tube will be addressed by a unique code offered by a computer software. The study will take place in community clinics and academic hospitals.

miR-17, miR-18b, miR-20a, miR-31, and miR-193a_3p, miR-125b, miR-345, miR-154, miR-409-3p, miR-127-3p, miR-214, miR-299-5p and miR-125b, miR-33a, miR-30e, miR-338-3p, miR-200a and miR-378 expression levels will be evaluated during multimodal therapy [1, 2, 52, 53].

### Plasma sample collection

Fifteen milliliters of whole blood samples are collected in Vacutainer tubes with spray-coated K2EDTA and stored at room temperature. Blood undergoes centrifugation for plasma separation within 2 h, to minimize the hemolysis and nucleic acid degradation.

Tubes are subjected within 1 h to a first centrifugation step at 2200×*g* for 15 min at room temperature. Plasma supernatants are transferred to 15-mL tubes, carefully avoiding contact with the lymphocytic ring, and tubes are centrifuged a second time at 3000×*g* and RT for 10 min to remove cellular debris.

Plasma samples are then collected into 1.5-mL cryovials, and all the aliquots are stored at − 80 °C.

### Plasma RNA extraction

Total RNA, including miRNAs, is isolated using a commercial kit (miRNeasy Mini Kit, Qiagen, Hilden, Germany) according to the manufacturer’s instructions. RNA concentration is assessed using a spectrophotometer. Adequate RNA concentration for mRNA expression is ≥ 30 ng/μL, and its quality is acceptable if the ratio between the value of the absorbance (*A*) at 260 nm and the absorbance at 280 nm is ≥ 1.8 and the ratio between the value of absorbance (*A*) at 260 nm and the one at 230 nm is ≥ 2.

### miRNAs expression assay

The nCounter miRNA Expression Assay (NanoString Technologies, Seattle, WA, USA) is designed to provide an ultra-sensitive, reproducible, and highly multiplexed method for detecting miRNAs in total RNA across all biological levels of expression. The assay provides a method for detecting miRNAs without the use of reverse transcription or amplification by using molecular barcodes called nCounter Reporter Probes. The assay can be run on total RNA isolated from liquid biopsy.

Sample preparation involves a multiplexed annealing of the specific tags to their target miRNA, a ligation reaction, and an enzymatic purification to remove the unligated tags. Sequence specificity between each miRNA and its appropriate tag is ensured by careful, stepwise control of annealing and ligation temperatures. Control RNA included in the nCounter miRNA Sample Preparation Kit allows the user to monitor the ligation efficiency and specificity through each step of the reaction.

NanoString technology is based on the direct molecular barcoding and digital detection of target miRNAs using a color-coded probe pair. The probe pair consists of a Reporter Probe, which carries the signal on its 5′ end, and a Capture Probe, which carries a biotin on its 3′ end. The complexity of the color codes, comprised of four colors in six positions, allows a large diversity of targets present in the same sample to be individually resolved and identified during data collection.

After hybridization, excess probes are washed away using a two-step magnetic bead-based purification.

Magnetic beads derivatized with short nucleic acid sequences that are complementary to the Capture Probe and the Reporter Probes are used sequentially. First, the hybridization mixture containing target/probe complexes is allowed to bind to magnetic beads complementary to sequences on the Capture Probe. Wash steps are performed to remove excess Reporter Probes and non-target cellular transcripts. After washing, the Capture Probes and target/probe complexes are eluted off the beads and are hybridized to magnetic beads complementary to sequences on the Reporter Probe. An additional wash is performed to remove excess Capture Probes. Finally, the purified target/probe complexes are eluted off the beads and immobilized on the cartridge for data collection. Data are analyzed using the nSolver™ software or other analysis programs.

### Primary endpoint

To evaluate the association of variation between preneoadjuvant and postneoadjuvant expression levels of miRNA with response to treatment.

### Secondary endpoints

- To evaluate the role of liquid biopsies in the monitoring of treatment
- To correlate changes in expression levels of miRNA following complete surgical resection with disease-free survival
- To evaluate the relation between changes in miRNA during surveillance and tumor relapse

### Data analysis

Patient subpopulation for the analysis is formed by the eligible patients with surgical evaluation and availability of plasma sample for the requested RNA analyses.

Baseline characteristics will be described for overall sample population by means of standard summary statistics (absolute frequencies, mean median and extreme values for continuous data, percentage for categorical data).

The association of variation between preneoadjuvant and postneoadjuvant expression levels of miRNA with treatment response will be presented with contingency tables and analyzed by mean of a logistic model. The odds ratio for the association and the AUC will be calculated, together with the corresponding 95% confidence intervals.

For secondary objectives (a) and (c), the role of liquid biopsies in the monitoring of treatment will be investigated by using a semi-parametric survival model (Cox model) with time-dependent variables, in order to incorporate modifications in the plasma measurements over time and their association with outcome, while for objective (b) the same analysis will be applied only on the subgroup of patients achieving pCR.

## Discussion

Mechanisms behind the recurrence/metastatic process in CRC are still not fully understood [52]. An important challenge in medical oncology is to identify patient or tumor characteristics to be correlated to the response to neoadjuvant and adjuvant treatment. Response variety of that implies an individualized treatment approach [52–54]. A new targeted approach to disease has been advocated for prevention and treatment based on individual characteristics regarding the environment, genes, lifestyle, and individual risk factors [4, 5, 53, 54].

MicroRNAs (miRNAs) are small, noncoding sequences that are post-transcriptional regulators of gene expression; depending on the genes they regulate, miRNAs can function as either oncogenes or tumor suppressors. In 2011, Della Vittoria Scarpati et al., showed their first results on miRNA evaluation in tissues as biomarkers for tumor response after neoadjuvant treatment on 38 patients. They found that two miRNAs (miR-630 and miR-622) were upregulated in all patients of group A (pathologic complete response) and downregulated in all patients of group B (all responses except complete) (sensitivity and specificity: 100%) [55].

In 2017, Jo et al. published their results of the analysis on circulant miRNA on 17 rectal cancer affected patients. All miRNAs that were retrieved from the group of upregulated miRNAs in the tumor showed a trend towards a reduced expression in the plasma of rectal cancer patients compared to the control samples. Expression levels of miRNAs in the plasma that were selected based on a decreased expression in the tumor compared to the mucosa were irregularly up- or downregulated miRNAs. They concluded that miR-30c and 31 may have a potential relevance as biomarker in rectal cancer to distinguish between cancer and non-cancer patients in the plasma [2]. As we stated before, based on findings by Jo et al., D’Angelo et al., and Yu et al., miR-17, miR-18b, miR-20a, miR-31, and miR-193a_3p, miR-125b, miR-345, miR-154, miR-409-3p, miR-127-3p, miR-214, miR-299-5p and miR-125b, miR-33a, miR-30e, miR-338-3p, miR-200a and miR-378 expression levels will be evaluated during multimodality therapy [1, 2, 51].

Comparing miRNA levels in all steps of the treatment with tumor response and finally with disease relapse and postoperative and oncologic outcome, we argue that miRNA could help to select patients who can or cannot benefit from surgery or from neoadjuvant alone in a setting of organ preservation, or from adjuvant treatment.

## Trial status

Protocol Version: V3.18 11/28/2018. Recruitment starting date: 3/25/2019. Recruitment ending date: 3/25/2022.

## Data Availability

Not applicable (the current manuscript does not contain any data related to patients; it is only a draft).

## Abbreviations

nCHT: Neoadjuvant chemoradiotherapy
TRG: Tumor regression grading
DNA: Deoxyribonucleic acid
RNA: Ribonucleic acid
miRNA: Microribonucleic acid
pCR: Pathologic complete response
CEA: Carcinoembryonic antigen
CRC: Colorectal cancer
ctDNA: Circulant deoxyribonucleic acid
RNase: Ribonuclease
RISC: RNA-induced silencing complex
